# Leptospirosis as Frequent Cause of Acute Febrile Illness in Southern Sri Lanka

**DOI:** 10.3201/eid1709.100915

**Published:** 2011-09

**Authors:** Megan E. Reller, Champika Bodinayake, Ajith Nagahawatte, Vasantha Devasiri, Wasantha Kodikara-Arachichi, John J. Strouse, Judith E. Flom, J. Stephen Dumler, Christopher W. Woods

**Affiliations:** Author affiliations: Johns Hopkins University School of Medicine, Baltimore, Maryland, USA (M.E. Reller, J.J. Strouse, J.S. Dumler);; University of Ruhuna, Galle, Sri Lanka (C. Bodinayake, A. Nagahawatte, V. Devasiri, W. Kodikara-Arachichi);; Johns Hopkins School of Public Health, Baltimore (J.E. Flom);; Duke University School of Medicine, Durham, North Carolina, USA (C.W. Woods)

**Keywords:** leptospirosis, serologic tests, Sri Lanka, fever, bacteria, acute febrile illness, Leptospira, research

## Abstract

To determine the proportion of fevers caused by leptospirosis, we obtained serum specimens and epidemiologic and clinical data from patients in Galle, Sri Lanka, March–October 2007. Immunoglobulin M ELISA was performed on paired serum specimens to diagnose acute (seroconversion or 4-fold titer rise) or past (titer without rise) leptospirosis and seroprevalence (acute). We compared (individually) the diagnostic yield of acute-phase specimens and clinical impression with paired specimens for acute leptospirosis. Of 889 patients with paired specimens, 120 had acute leptosoirosis and 241 had past leptospirosis. The sensitivity and specificity of acute-phase serum specimens were 17.5% (95% confidence interval [CI] 11.2%–25.5%) and 69.2% (95% CI 65.5%–72.7%), respectively, and of clinical impression 22.9% (95% CI 15.4%–32.0%) and 91.7% (95% CI 89.2%–93.8%), respectively. For identifying acute leptospirosis, clinical impression is insensitive, and immunoglobulin M results are more insensitive and costly. Rapid, pathogen-based tests for early diagnosis are needed.

## Abstract 2

TOC summary: Clinical impression and serologic tests of acute-phase specimens are insensitive, and rapid, pathogen-based tests are needed.

Leptospirosis is an endemic zoonosis in the tropics, where a favorable climate enables the pathogenic spirochete *Leptospira interrogans* to survive in the environment (*1*). Furthermore, many tropical residents have repeated direct and indirect exposures to infected animals that excrete leptospires in their urine (*2*).

Sri Lanka, with a rapidly growing population of ≈20 million, has a reported annual incidence of leptospirosis of 5.4 cases/100,000 persons, the sixth highest incidence worldwide (*3*). Approximately 28% of Sri Lanka’s workforce is employed in agriculture, and reported cases of leptospirosis fluctuate with rainfall and farming cycles. Historically, ≈200 cases per million population per year have been reported from the southern and north–central regions, where the disease is hyperendemic (*3*). However, incidence rates are imprecise estimates because leptospirosis is easily confused with undifferentiated fever of other causes (*1*), and few cases are laboratory confirmed (*4*). In the past 2 decades, clinical cases have been increasingly reported (*5*), including >7,000 cases in 2008 (*6*).

To determine the prevalence of acute and past leptospirosis in southern Sri Lanka, assess tools for acute diagnosis, and identify associated features, we collected epidemiologic and clinical data and paired serum specimens from a prospective cohort of children and adults with undifferentiated fever. The institutional review boards of the University of Ruhuna, Johns Hopkins University, and Duke University Medical Center approved this study.

## Materials and Methods

### Study Participants

We recruited patients in the emergency department, acute care clinics, and adult and pediatric wards of the Karapitiya Teaching Hospital in Galle, the largest (1,300-bed) hospital in southern Sri Lanka, during March–October 2007. We enrolled consecutive febrile (38°C, tympanic) patients >2 years old without antecedent (≤7 days) trauma or hospitalization who sought treatment during clinic hours (8:00 am–4:00 pm Monday–Friday, and 8:00 am–2:00 pm Saturday). Study doctors verified patient eligibility and willingness to return for follow-up and obtained written informed consent from patients (>18 years of age) or parents (of those <18 years of age) and assent from those 12–17 years of age.

Study personnel recorded structured epidemiologic and clinical data, including duration of illness and the clinical provider’s presumptive diagnosis, on a standardized form. Study doctors then obtained blood for on-site clinician-requested testing and subsequent off-site research-related testing. Patients returned for clinical and serologic follow-up 2–4 weeks later or were visited at home if they were unable to return and could be located. Blood was centrifuged, and serum specimens were frozen on site at −80°C, shipped on dry ice, and thawed only when separated into aliquots and when tested.

### Serologic Testing

We tested paired serum specimens for the presence of specific *Leptospira* immunoglobulin (Ig) M by ELISA (Institut Viron Serion GmgH, Warburg, Germany), according to the manufacturer’s instructions. Briefly, rheumatoid factor (RF)-absorbent was first diluted 1:4 in buffer. Serum specimens from patients and controls were then diluted (1:100) in RF-absorbent buffer to accomplish removal of IgM RF, transferred to antigen-coated microtest wells, and incubated at 37°C for 60 min. After wells were washed with phosphate-buffered saline, antihuman IgM (conjugated to alkaline phosphatase and *p*-nitrophenylphosphate) was added. After incubation of the wells for 20 min, sodium hydroxide was added to each well to stop the reaction, and the absorbance at 405 nm was measured.

The ELISA provided qualitative results—positive, negative, and equivocal (borderline positive/negative). Using a standard curve and evaluation table provided with the kit, we obtained the optical density measurements, which were adjusted for plate-to-plate variation with a correction factor and gave quantitative results that correlated with titers (*7*).

### Case Definitions

Acute leptospirosis was defined as definitive seroconversion (negative acute-phase serum specimen to positive convalescent-phase serum specimen) or the equivalent of a 4-fold rise in IgM titer. We excluded from analyses of acute leptospirosis specimens with equivocal IgM test results or those lacking a convalescent-phase sample.

Past leptospirosis was defined as stable or decreasing IgM titers. We excluded from analyses of past leptospirosis specimens with equivocal IgM test results or those lacking a convalescent-phase sample. IgM seroprevalence was defined as the prevalence of leptospirosis by *Leptospira* IgM in acute-phase serum specimens, independent of whether a convalescent-phase specimen was obtained or its result.

### Statistical Analysis

Proportions were compared by the χ^2^ test or Fisher exact test and continuous variables by Student *t* test or the rank sum test if distribution was not normal. Confidence intervals (CIs) for risk ratios were calculated by exact methods. We assessed IgM in the acute-phase sample for seroprevalence and clinical impression was compared with results of paired-serum specimen testing for acute leptospirosis. We specifically correlated epidemiologic features, duration of illness, and symptoms and signs with serologic test results. Analyses were performed with Stata IC 11.0 (StataCorp LP, College Station, TX, USA).

## Results

### Patient Characteristics

Paired serum specimens were available from 889 (82.4%) of 1,079 patients consecutively enrolled. Among those, a diagnosis of acute leptospirosis could be confirmed or refuted for 773 (87.0%) of 889, because serologic results were inconclusive for 116. The likelihood of a participant’s returning for convalescent-phase serum sampling and clinical follow-up did not differ by age (p = 0.10). Female patients were slightly more likely to return for follow-up (85.8 vs. 80.6%; p = 0.03). Most (90.2%) patients lived in rural areas and were more likely to return for follow-up than were those who lived in urban areas (83.5 vs. 71.4%; p = 0.002). The proportion with secondary education was similar in the 2 groups (21.7 vs. 19.6%; p = 0.51), as was reported duration of fever and of illness (p = 0.15 and p = 0.13, respectively).

Of the 773 patients with conclusive serologic results, the median age was 30.1 years (interquartile range [IQR] 19–47 years). More patients were male (60.6%) than female, and the median age did not differ by sex (p = 0.78). The median reported duration of fever and of illness was 3 days (IQR 2–5 days and 2–7 days, respectively). Many (37.6%) reported taking an antimicrobial drug before seeking treatment. The median interval between acute-phase and convalescent-phase follow-up was 21 days (IQR 15–33 days).

### Diagnosis of Acute Leptospirosis

**Figure 1:**
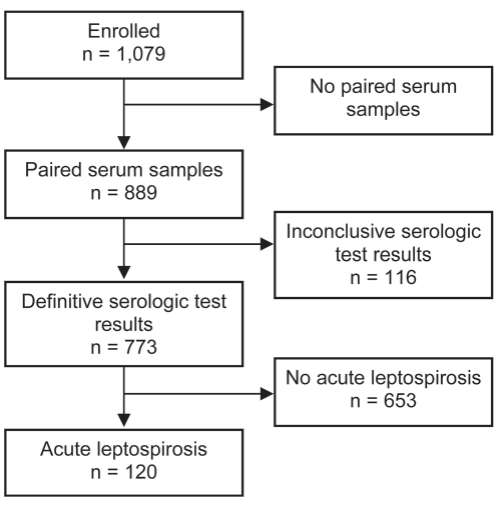
Flowchart indicating selection of study participants with a diagnosis of acute leptospirosis, southern Sri Lanka, 2007.

Acute leptospirosis was confirmed for 120 patients (Figure 1): by seroconversion for 96 patients and by a 4-fold rise in titer for 24 patients (in 21 acute-phase specimens with positive results and in 3 acute-phase specimens with equivocal results); acute leptospirosis was excluded for 653 patients. Data on presumptive clinical diagnosis were available for 714 patients, including for 109 of 120 with acute leptospirosis. Of these patients, 25 received a correct diagnosis of acute leptospirosis, and 84 received an incorrect diagnosis of another disease. The sensitivity and specificity of clinical impression were 22.9% (95% CI 15.4%–32.0%) and 91.7% (95% CI 89.2%–93.8%), respectively. Finally, 279 patients were seropositive at enrollment, including 201 with past leptospirosis, 40 with possible recent leptospirosis (second specimen equivocal), 21 with acute leptospirosis, and 57 without paired serum specimens. Therefore, if acute-phase IgM had been used to diagnose acute leptospirosis instead of paired serum specimens, only 21 of 120 acute infections would have been identified (sensitivity 17.5%, 95% CI 11.2%–25.5%), and 201 of 653 patients without acute leptospirosis would have been given an erroneous diagnosis (specificity 69.2%, 95% CI 65.5%–72.7%). Thus, IgM seropositivity at the acute-phase visit correlated poorly with acute leptospirosis.

### Demographic Features of Patients with Acute or Past Leptospirosis

**Figure 2:**
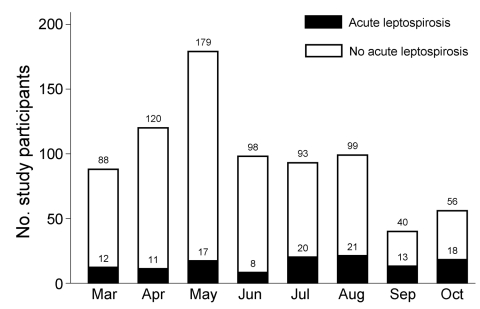
Leptospirosis cases by month among study patients enrolled with fever, southern Sri Lanka, 2007

**Figure 3:**
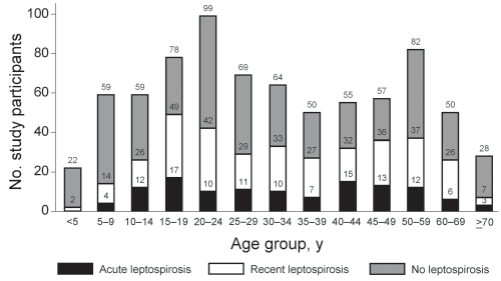
Age distribution of study patients enrolled with fever, southern Sri Lanka, 2007

The demographic characteristics of patients are listed in Table 1. Those with acute or past leptospirosis were older (median 32 years vs. 27 years; p = 0.01) than those without acute or past leptospirosis. Reporting exposure to paddy fields (relative risk [RR] 1.9, 95% CI 1.6–2.1; p<0.0001) and working as a farmer (RR 1.9, 95% CI 1.6–2.3; p = 0.0001) were strongly associated with acute and past leptospirosis. Reporting no fresh water exposure and boiling drinking water were protective against leptospirosis. Patients enrolled during March–June were less likely to have acute leptospirosis than those enrolled during July–October (Figure 2). Children <10 years of age were much less likely to have acute or past leptospirosis; in contrast, leptospirosis was more common in older adolescents and young adults (Figure 3).

**Table 1:** Demographic characteristics of febrile patients with acute or past leptospirosis versus those who had neither acute nor past leptospirosis, southern Sri Lanka, 2007*

Demographic characteristic	% With acute or past leptospirosis, n = 361	% With neither acute nor past leptospirosis, n = 412	p value
Median age, y (IQR)	32 (20–46)	27 (16–47)	0.02
Male sex	60	64	0.14
Residence			
Urban	8	9	0.88
Rural	92	91	
Type of work			<0.0005
Home	27	25	
Laborer	26	21	
Farmer	6	1	
Merchant	2	4	
Student	20	25	
Other	20†	24	
Animal exposures			
Dog	57†	54	0.43
Rodent	27	30	0.35
Cow	7	4†	0.13
Swim/bathe/wade			
None	66	82	<0.0005
River	14	11	
Paddy field	19	4	
Other	2‡	3	
Water source			0.001
Tap	31	33	
Boiled	6	14	
Well	63	52	
Other	0.3	1	

*IQR, interquartile range. †Does not add to 100% due to rounding. ‡Adds to >100% due to multiple exposures.

The median duration of illness at hospital visit for those with acute leptospirosis diagnosed by seroconversion was 3 days (IQR 2–5 days), and for those with acute leptospirosis diagnosed by a 4-fold rise in titer, 4 days (IQR 3–5 days; p = 0.09). The median interval between serum sampling was 22 days (IQR 15–31 days). The follow-up time was slightly longer for those with leptospirosis diagnosed by 4-fold change in titer than for those with diagnosis by seroconversion (median 26 vs. 20 days; p = 0.08). The median age was 33.6 years (IQR 18.7–45.6 years), and more patients were male (69.2%) than female (p = 0.07).

### Clinical Features of Acute Leptospirosis

Clinical features associated with acute leptospirosis are listed in Table 2. Headache was the most frequent (≈80%) symptom reported; lethargy, muscle pain, and joint pain were also reported by >50% of patients. Lethargy and cough were reported less often in patients with acute leptospirosis, and oliguria, dysuria, and muscle and joint pain were reported more often. Patients with acute leptospirosis were more likely to have conjunctival suffusion (RR 2.4, 95% CI 1.7–3.4; p<0.0001) and less likely to have pharyngeal exudates. Abdominal tenderness and hepatomegaly were slightly more frequent in those with acute leptospirosis. Jaundice, splenomegaly, arthritis, rash, and meningismus were uncommon in both groups. Patients with acute leptospirosis had similar leukocyte counts to patients who did not, and slightly lower hemoglobin concentrations and platelet counts and lower absolute lymphocyte counts. A greater proportion of patients with acute leptospirosis were admitted to the hospital (84.2% vs. 70.1%) than were others with fever (p = 0.002), but they did not have a longer stay (median 4 days, IQR 3–6 days; p = 0.83). At the convalescent-phase follow-up visit, patients with acute leptospirosis reported a longer total duration of fever than others (5 days [IQR 3–7 days] vs. 4 days [IQR 3–6 days]; p = 0.008). No one with confirmed acute leptospirosis died, but most (11 of 12) deaths occurred before follow-up. Among those who died, the acute-phase serum specimen was IgM-negative for 8 patients, IgM-positive for 2, and results were equivocal for 1.

**Table 2:** Clinical characteristics of febrile patients with acute leptospirosis versus those without acute leptospirosis, southern Sri Lanka, 2007*

Clinical characteristic	With acute leptospirosis, n = 120	Without acute leptospirosis, n = 653	p value
Symptom			
Headache	81	78	0.63
Sore throat	28	29	0.96
Cough	44	60	<0.005
Dyspnea	15	18	0.48
Joint pain	56	43	<0.01
Muscle pain	62	46	<0.005
Lethargy	58	70	<0.01
Abdominal pain	22	18	0.41
Emesis	45	37	0.10
Diarrhea	13	12	0.80
Dysuria	20	13	<0.05
Oliguria	17	8	<0.005
Sign			
Mean temperature, °C (SD)	38.6 (0.6)	38.5 (0.6)	0.17
Median heart rate, beats/min (IQR)	80 (72–100)	84 (76–96)	0.85
Mean body mass index, kg/m^2^ (SD)	20.7 (4.8)	19.6 (5.1)	<0.05
Conjunctival suffusion	29	12	<0.0005
Pharyngeal exudate	8	15	<0.05
Lymphadenopathy	24	23	0.73
Jaundice	2	2	0.73
Lung crackles	10	14	0.27
Tender abdomen	13	9	0.21
Hepatomegaly	8	5	0.31
Laboratory parameter, median (IQR)			
Leukocytes, cells/μL	7,800 (5,700–10,500)	7,900 (5,600–11,300)	0.52
Absolute neutrophil count, cells/μL	5,530 (3,854–8,424)	5,313 (3,344–7,952)	0.49
Absolute lymphocyte count, cells/μL	1,638 (1,210–2,574)	2,140 (1,541–2,856)	<0.005
Hemoglobin, g/dL	12.3 (11.6–13.5)	12.6 (11.7–13.8)	<0.05
Platelets, × 1,000/μL	200 (164–256)	231 (190–289)	<0.0005

*Values are % patients in that category (with vs. without leptospirosis), except as indicated. IQR, interquartile range.

## Discussion

We found that leptospirosis was a common, but often clinically unsuspected, cause of fever among unselected patients seeking care in southern Sri Lanka. Farming and rice paddy work were associated with increased risk for leptospirosis, as was exhibiting acute febrile illness during the harvesting season (July–October). In our setting, testing acute-phase serum specimens alone for IgM was less sensitive and specific for diagnosing acute leptospirosis than was diagnosis by observation of clinical features.

Isolation of *Leptospira* spp. confirms acute infection, but requires special media that must be incubated for up to 13 weeks, and has low sensitivity (*1*). Therefore, the diagnostic standard for acute leptospirosis is a definitive rise in titer between paired serum specimens (*1*). Historically, these results have been obtained by the microscopic agglutination test (MAT). Serum specimens are first reacted with live antigen suspensions of different leptospiral serovars. After incubation, the serum-antigen mixtures are examined and titers determined. For paired serum specimens, the highest dilution of serum at which 50% agglutination occurs must be determined, a laborious and inherently subjective task (*1*).

Sensitivity is compromised if all locally relevant serovars are not represented, and live cultures of all serovars tested must be maintained whether live or formalin-killed antigens are used. Subculturing many *Leptospira* spp. weekly is hazardous for personnel, and laboratory-acquired infections occur (*1*). Reading a MAT requires a dark-field microscope, which is unavailable in most laboratories, including Karapitya Teaching Hospital. Furthermore, a MAT detects both IgM and IgG and lacks sensitivity and specificity when early acute-phase serum specimens alone are tested rather than paired specimens (*1*). Patients with fulminant illness may die before seroconversion occurs. A MAT may also be less sensitive than an IgM ELISA, even for convalescent-phase specimens. Relative to isolation of *Leptospira* spp., the reported sensitivities of MATs for acute, late acute-phase, and convalescent-phase serum specimens were 30%, 63%, and 76%, respectively, and of IgM ELISA, 52%, 89%, and 93%, respectively, in 1 study in Barbados (*8*). In another study, results of MAT and IgM ELISA for a single early acute-phase specimen were comparable (49%) (*9*).

To overcome the practical pitfalls of MATs, we chose to test paired serum specimens by IgM ELISA, which requires only an inexpensive plate reader, is relatively easy to perform, and provides objective, reproducible results as demonstrated by a parallel comparison of results of multiple commercial assays (*9*). Furthermore, results of performing IgM ELISA on paired serum specimens from patients from various geographic regions have compared favorably (sensitivity 86.5%, specificity 97.0%) with MAT results (*10*). These data suggest that at least 13.5% of febrile illnesses in our cohort were acute leptospirosis. We assayed paired specimens for IgM instead of IgG, because the kinetics of IgG are more variable (*11**,**12*). IgM generally appears within 1 week of symptoms and persists for months to years after infection (*13*) with titers higher than those of IgG throughout (*11**,**14*). Additionally, in some patients for whom leptospirosis is confirmed by culture and MAT, IgG never develops (*11**,**13*).

We chose a commercially available IgM ELISA that has performed comparably to others in detecting serovars likely present in southern Sri Lanka (*9*). IgM is inherently cross-reactive, and thus serovars themselves are not detected. In a recent study from central Sri Lanka, the predominant serovars were Mednensis and Hardjo, but others included Australis, Ballum, Canicola, Celledoni, Cynopteri, Pomona, and Robinsoni (*15*). Previously, identification of the serovar Icterhemorrhagiae in Sri Lanka led to control of the rodent vector (*16*). The assay we used has reliably reacted with serovars Icterohemorrhagiae, Canicola, Grippotyphosa, Bataviae, Pomona, Tarassovi, Copenhageni, Bratislava, Hebdomadis, Sejroe, Australis, Panama, Pyrogenes, Patoc, Hardjo, and Cynopteri (*7**,**17*).

Notably, detection of acute-phase IgM did not predict which patients had acute leptospirosis, despite its widespread use as an acute diagnostic test. Retrospective studies suggest sensitivities and specificities of 36%–53% for single acute-phase IgM and 90%–99% for MAT on paired serum specimens, respectively (*9**,**10*). The varied sensitivity likely reflects different case definitions and control groups, timing of acute-phase specimen collection (up to 42 days after onset), geography and serovar distribution, platforms and protocols (e.g., ELISA ± use of RF absorbent, indirect hemagglutination, and dot-ELISA and IgM dipsticks), and convenience sampling. Notably, ELISA of single (acute-phase) serum specimens has performed as well or better than MAT or indirect hemagglutination of single serum specimens, so those strategies are not advised (*10*).

The most widely recognized problem with using acute-phase IgM to identify acute leptospirosis is that many persons in disease-endemic areas are expected to have preexisting antibodies. Some have advocated higher cut-offs to discriminate between acute infection and preexisting antibodies (*1*), because patients may be harmed as much by incorrectly attributing fever to leptospirosis as by falsely excluding it. However, data to support this approach are lacking, and misclassification could occur both early in acute infection (impaired sensitivity because antibody is not yet present) and later (impaired specificity because antibody is persistent). In our rigorous comparison of single vs. paired serum specimens, we found acute-phase IgM had especially poor sensitivity (17.5%), since patients sought treatment early (≈3 days), and more acute infections were identified by seroconversion than by a definitive rise in titer. The median duration of illness in those diagnosed by rise in titer versus seroconversion tended to be longer (4 days [IQR 3–5 days] vs. 3 days [IQR 2–5 days], respectively; p = 0.09). Requiring a higher cutoff titer would further impair sensitivity. Hence, acute-phase IgM testing alone has multiple limitations for diagnosis of acute leptospirosis, regardless of the cut-off.

Only a few studies have evaluated the use of serologic testing for identifying leptospirosis in febrile cohorts. In Laos, 372 febrile patients were evaluated with ELISA (Panbio Ltd., Brisbane, Queensland, Australia) and immunochromatographic testing (ICT), which was compared with the MAT; acute leptospirosis (single titer >400 or 4-fold rise in titer) was identified in 23 (12.4%) of 186 patients (*18*). The sensitivity of ELISA and ICT was relatively high (60.9% and 47.3%, respectively), which could be explained by a long duration of fever (median 9 days). The sensitivity of ELISA for acute-phase versus convalescent-phase serum specimens was comparable (60.9% and 65.2%, respectively), but convalescent-phase serum specimens were obtained 4.5 days after acute-phase serum specimens. The specificity of both assays was similarly poor (65.6% for ELISA and 75.5% for ICT). In Thailand, Cohen et al. identified acute leptospirosis in 67 (9.5%) of febrile subjects using 2 rapid assays, a dipstick, and latex slide agglutination test (*19*). Patients sought treatment after a mean of 3.4 days of fever and returned 22 days later. Compared with MAT on paired serum specimens, the sensitivity of testing acute-phase serum with the dipstick and latex slide agglutination tests was 22% and 13%, respectively, which is similar to our findings.

Strengths of our study include the rigorous, prospective design, uniquely large sample size, inclusion of an unstudied population believed to be at high risk, an unusually high rate of follow-up, and clinical correlation. To minimize selection bias, we used standardized criteria to sequentially enroll a large cohort (≈900 patients) with thorough follow-up to enable assessment of acute-phase IgM testing versus clinical impression and relevant epidemiologic and clinical features. Those patients from whom paired serum specimens were not available differed only slightly from the included population. We excluded the few with equivocal results to avoid possible misclassification with resultant potential failure to identify significant predictive clinical features. By rigorously distinguishing acute from recent leptospirosis, we were able to confirm that myalgias and arthralgia were frequent symptoms and that the presence of conjunctivitis or conjunctival suffusion is diagnostically helpful. Leptospirosis in this cohort was relatively mild, as evidenced by stable vital signs, the absence of jaundice, and complete blood counts within nearly normal ranges. The sparse laboratory data reflect standard clinical practice in which automated testing is largely unavailable in the public sector, expensive in the private sector, and thus infrequently obtained.

Our results might have differed if we had used a different diagnostic standard, but culture, for example, is insensitive, labor intensive, unlikely to be available soon at Karapitya Teaching Hospital and many similar hospitals, and too slow to guide clinical management. We may have misclassified the number of days with fever, since temperatures are infrequently taken at home or in the hospital; however, duration of fever correlated well with that of symptoms, and no systematic bias would be expected, since etiologic diagnoses were not known when patients sought treatment at the hospital. Our estimate of leptospirosis may be low if a wider array of serovars is circulating in southern Sri Lanka than were detected by the ELISA used; however, no other available commercial assay would have been expected to be more sensitive.

We conclude that leptospirosis causes substantive illness in southern Sri Lanka. Furthermore, we found that testing acute-phase serum specimens for IgM has multiple limitations for the diagnosis of acute leptospirosis, because a positive result more often denoted past infection than an acute infection, and results were negative early in infection. Clinical impression is comparatively better without added cost (*20*). Paired serum specimens can provide rigorous diagnosis, but patients and clinicians need rapid diagnosis to guide clinical management. A few antigen-based or nucleic acid–based rapid tests have been described, but prospective clinical validations are limited (*17**,**21**–**25*). Rapid, pathogen-based tests for early diagnosis need to be developed.
